# Vital Sign Monitoring Using FMCW Radar in Various Sleeping Scenarios

**DOI:** 10.3390/s20226505

**Published:** 2020-11-14

**Authors:** Emmi Turppa, Juha M. Kortelainen, Oleg Antropov, Tero Kiuru

**Affiliations:** VTT Technical Research Centre of Finland Ltd., P.O. Box 1300, 33101 Tampere, Finland; juha.m.kortelainen@vtt.fi (J.M.K.); oleg.antropov@vtt.fi (O.A.); tero.kiuru@vtt.fi (T.K.)

**Keywords:** biomedical monitoring, biomedical signal processing, contactless, health monitoring, heart rate, heart rate variability, millimeter wave radar, respiratory rate

## Abstract

Remote monitoring of vital signs for studying sleep is a user-friendly alternative to monitoring with sensors attached to the skin. For instance, remote monitoring can allow unconstrained movement during sleep, whereas detectors requiring a physical contact may detach and interrupt the measurement and affect sleep itself. This study evaluates the performance of a cost-effective frequency modulated continuous wave (FMCW) radar in remote monitoring of heart rate and respiration in scenarios resembling a set of normal and abnormal physiological conditions during sleep. We evaluate the vital signs of ten subjects in different lying positions during various tasks. Specifically, we aim for a broad range of both heart and respiration rates to replicate various real-life scenarios and to test the robustness of the selected vital sign extraction methods consisting of fast Fourier transform based cepstral and autocorrelation analyses. As compared to the reference signals obtained using Embla titanium, a certified medical device, we achieved an overall relative mean absolute error of 3.6% (86% correlation) and 9.1% (91% correlation) for the heart rate and respiration rate, respectively. Our results promote radar-based clinical monitoring by showing that the proposed radar technology and signal processing methods accurately capture even such alarming vital signs as minimal respiration. Furthermore, we show that common parameters for heart rate variability can also be accurately extracted from the radar signal, enabling further sleep analyses.

## 1. Introduction

Monitoring vital signs is routine practice to detect patient deterioration at healthcare facilities. Changes in vital signs can indicate serious medical problems, and catching the early signs may improve survival rates for the relevant conditions [1]. Lately, the general population has become more interested in self-monitoring, which has provoked the emergence of numerous commercial wearable devices, particularly ones specialized in heart rate monitoring. Such wearable devices have also been examined in the context of monitoring healthcare patients [2,3]. Yet, wearable and other attachable devices can cause eczema and they depend on a sufficient contact to operate. In contrast, remote monitoring is contactless, unobtrusive, and could monitor several vital signs simultaneously while providing more user-friendly monitoring in various environments [4,5,6,7]. Remote monitoring with radar technology could reform sleep monitoring at home and nursing homes by removing the often disturbing tactile sensation of a wearable device and the wired sensors that tend to detach. It can also be a cost-effective solution as it does not require disposable elements such as electrodes. Ultimately, remote measurements could ease monitoring in critical care taking place in hospitals [8,9].

Periodic variations in the measured radar signal, which are caused by micromotions on the body surface, can convey information regarding the two vital signs considered herein: heart rate and respiration rate. In this paper, we study a frequency modulated continuous wave (FMCW) radar developed at VTT Technical Research Centre of Finland [6,10]. Similar millimetre wave chipsets and development boards capable of time domain multiplexing are also available commercially [11,12]. The radar transmits frequency-modulated electromagnetic waves and can detect the phase of the received signal with about one degree accuracy. Its high resolution enables the detection of microscopic vascular pulsations on the skin.

Prior studies on FMCW radars have already established the potential for vital sign monitoring applications [4,13,14,15]. Among the heart rates extracted by Anitori et al. 60% were within 10% of their reference values [13]. Alizadeh et al. later achieved 94% and 80% accuracies for respiration rate and heart rate, respectively [15]. However, while both studies examined monitoring in lying positions, Alizadeh et al. included only one subject as opposed to the six participants by Anitori et al. Additionally, both studies used a commercial, non-medical device for reference signals. In contrast, Wang et al. and Adib et al. studied ten or more subjects in seated positions, using medical devices for reference [4,14]. Wang et al. reported approximately 5-31% and 11-20% relative errors for respiration and heart rate, respectively, depending on the exact position [14]. Adib et al. demonstrated median accuracies of 99.4% and 99% for respiration and heart rate, respectively, and were also able to measure multiple targets simultaneously [4].

In this study, we explore the potential of FMCW radar technology for the special application of nocturnal vital sign monitoring by emulating diverse real-life scenarios. Unlike previous studies, we pursue a wide range of both heart and respiration rates to discuss the applicability of FMCW radars to monitor people with different conditions. Capturing a wide range of vital signs is essential for sleep analysis and for monitoring sleep disorders, such as hypopnoea, and for following the effects of possible interventions [16,17,18]. Whereas previous works have established suitable accuracies for commercial use at home and office environments, we demonstrate the applicability of an FMCW radar in the aforementioned clinical applications by showing that it can accurately capture even alarmingly anomalous vital signs, such as shallow respiration.

We include ten volunteers in our study in order to account for the natural differences between individuals and to ensure a level of robustness in our vital sign extraction methods [19]. The participants are monitored in varying lying positions while performing simple activities, emulating vital sign variations of real-life sleeping scenarios. We extract their interbeat intervals (IBI) and respiratory rates, and compare to reference data acquired using Embla Titanium, a certified medical device.

Despite the deliberately challenging study setting, we are able to surpass the results of previous studies in heart rate monitoring accuracy. Moreover, we establish high accuracy in respiration monitoring even with minimal respiratory motion, which we expect to promote radar-based monitoring in clinical settings. We provide further grounds for such clinical applications by demonstrating, for the first time to our knowledge, accurate radar-based extraction of features commonly used in heart rate variability (HRV) analysis, an essential tool in modern stress monitoring applications [20,21,22,23].

## 2. Materials and Methods

**Figure 1:**
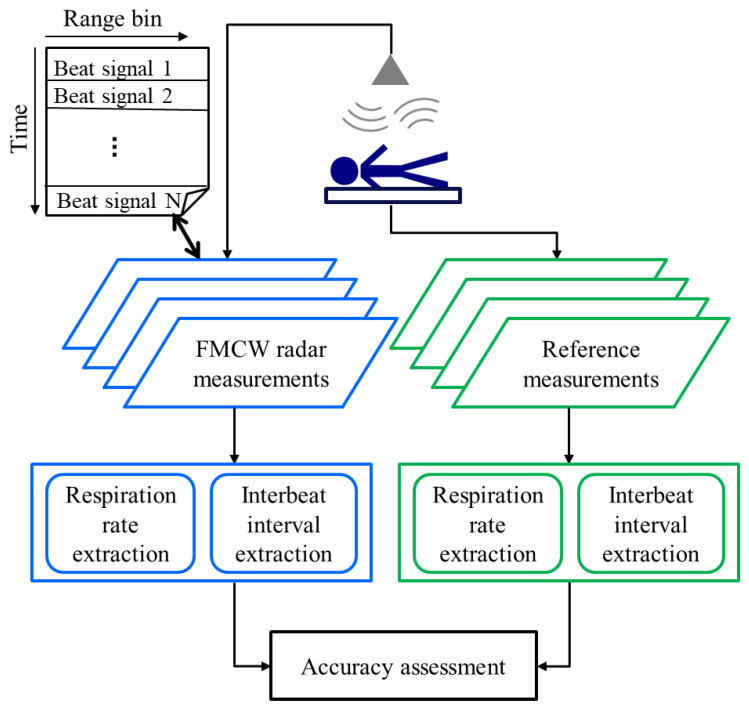
The overall measurement and evaluation workflow. The radar and reference data are processed separately. A beat signal describes the difference between the transmitted and received signals.

The workflow of our study is schematically illustrated in Figure 1. In this section, we elaborate on each item step-by-step. We used an in-house developed FMCW radar (Section 2.1) and reference devices (Section 2.2) to measure each participant in the study group (Section 2.3) during a set of activities resembling real-life sleeping scenarios (Section 2.4). The measured data were analysed using robust state-of-the-art approaches to extract interbeat interval, heart rate, and heart rate variability parameters, as well as respiration rate (Section 2.5, Section 2.6 and Section 2.7). Finally, the accuracy assessment of the retrieved estimates was performed using the methods described in Section 2.8.

### 2.1. Frequency Modulated Continuous Wave Radar

**Figure 2:**
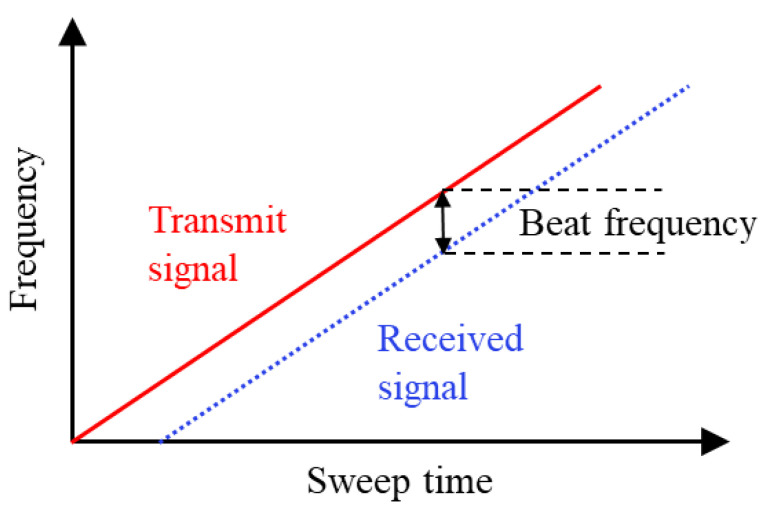
Illustration of the frequency modulated continuous wave (FMCW) radar principle [6]. Using a frequency sweep allows to compute the distance from the radar to the target.

An FMCW radar transmits a frequency-modulated continuous signal and detects its reflection. As visualized in Figure 2, the distance to an object can be computed based on the beat frequency, i.e., the frequency difference between the transmitted and received signals [24].

An FMCW radar measurement can be divided into chirps, or frequency sweeps, where the transmit signal frequency modulates (or sweeps) through the specified frequency band [10]. The instantaneous profile of the observed distances, also known as the complex range profile, can be extracted from the collected samples of beat signals by applying a Fast Fourier Transform (FFT) to each set of samples from the same chirp. Using the resulting set of complex FFTs, the beat signal amplitude and phase can be extracted for the desired range bin. Furthermore, the complex range profiles from consecutive chirps can be stacked into the range slow-time matrix, which contains the phase information of the beat signal as a function of time, the main signal needed for vital sign extraction.

The in-house FMCW radar used in this study operated at the carrier frequency of 24 GHz with a 250 MHz bandwidth. The radar has a range resolution of 60 cm, micromotion detection accuracy below 1 µm, and receiver noise figure of 12 dB. In this study, we explore two chirp repetition frequencies, i.e., sampling frequencies: 110 Hz and 154 Hz. While the maximum operable frequency of 154 Hz can capture more detailed information, the lower one is more stable to operate with the existing software. Thus, we take the opportunity to examine whether lowering the sampling frequency deteriorates performance in vital sign monitoring.

**Figure 3:**
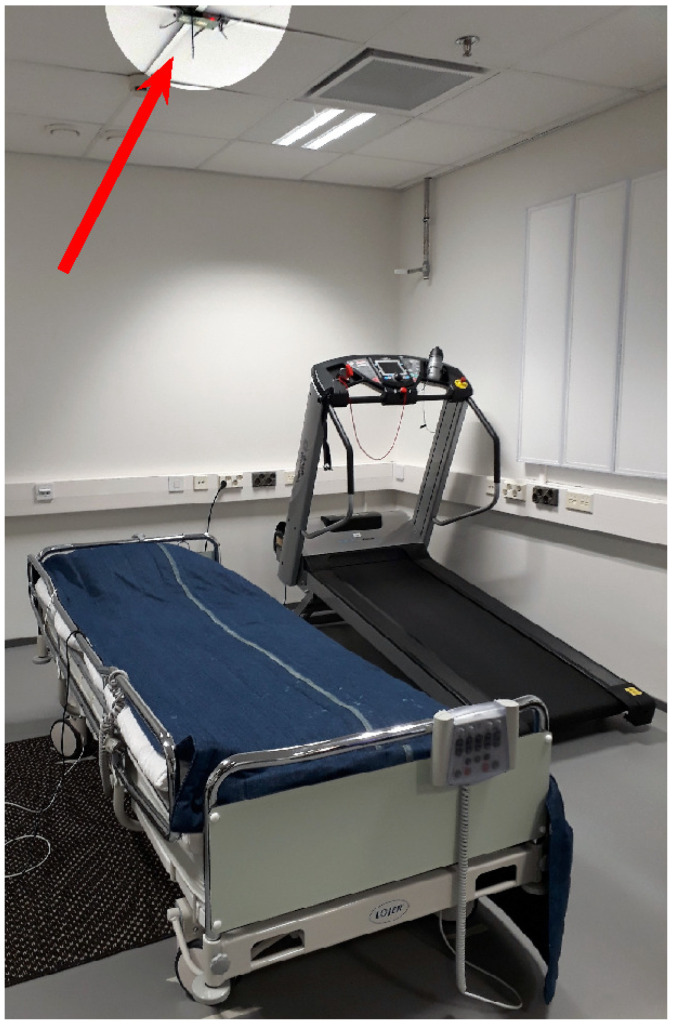
The measurement setting. The FMCW radar (highlighted in brighter tones and pointed out with a red arrow) is mounted on the ceiling above the bed. The treadmill beside the bed was used for exercising during the measurement session.

The radar was mounted on the ceiling above a bed, facing downwards towards the subject above the torso, at a fixed distance of about 2 m, as portrayed in Figure 3. The radar antenna 3 dB beam width was $65^{\circ }$ along the length of the bed and $26^{\circ }$ along the perpendicular direction. The field of coverage was configured to 3 m to reduce noise.

### 2.2. Reference Devices

Reference signals were collected simultaneously with the radar data, using the Embla titanium portable polysomnography (PSG) system, a CE certified class II device in use in many physiological studies worldwide [25]. Two electrocardiographic (ECG) electrodes were attached to the subject to collect the reference ECG signal at 256 Hz sampling frequency. One electrode was attached under the right-side collarbone and the other on the lower left part of the thoracic cage. One respiratory inductive plethysmography (RIP) belt on the thorax was used to collect the reference respiration signals at 32 Hz sampling frequency.

Because of technical issues that sometimes occur in the measurements and downgrade the signal-to-noise ratio, an additional reference was collected at 110 Hz using VTT’s ballistocardiography (BCG) based sensor sheet installed beneath the mattress topper. The sensor sheet can detect respiration rate with 1.5% error relative to RIP belts [26].

### 2.3. Study Group

We measured eleven participants from age 25 to 55 (37 on average, 2 female), who signed an informed consent form prior to the measurement, after receiving information about the measurement protocol and the study objectives. The study did not intervene with the physical integrity of the volunteers and the study setting was not harmful or otherwise disturbing.

### 2.4. Measurement Protocol

As presented in Table 1, the measurement protocol was a combination of three distinct activities, each measured for two minutes at a time: relaxed respiration, hypopnoea simulation, and recovering after physical exercise. The hypopnoea simulation comprised one minute of shallow respiration and another minute of normal respiration. These sub-activities are presented separately in Table 1 for clarity. Before the final activity of recovering after exercise, the participants walked on a treadmill with roughly 15% inclination at 2 km/h, for two minutes. The participants were not measured during the exercise, and they were allowed to interrupt at any time. Nevertheless, all participants exercised the full two minutes.

The relaxed respiration and hypopnoea simulation activities were measured once in all four different positions: supine, left lateral recumbent, right lateral recumbent, and prone. This was true with one exception: the relaxed respiration activity was repeated in the supine position to ease the participant’s transition to the next activity. The final activity (after the exercise) was only measured in the supine position. The participants were given sufficient transition time for each change of position.

The protocol was repeated with two sampling frequencies using a reduced protocol with the more unstable 154 Hz sampling frequency (see Table 1). Measurement segments using the different sampling frequencies were performed sequentially but in alternating order between participants. In total, 32 min of activity data were collected per participant (20 min with 110 Hz and 12 min with 154 Hz). This comprises 14 min in the supine position and 6 min in each of the three other positions.

### 2.5. Heart Rate Extraction

The interbeat interval was extracted from the reference and radar devices using different methods (see Figure 1). The R-to-R interval, used as reference IBI, was extracted from the ECG signal using the findpeaks function by MATLAB^®^ (minimum peak distance 0.3 s) after trend removal. To extract IBI from the radar signal, cepstral analysis, a variant of spectral analysis, was applied [27]. It is able to emphasize the significantly small heartbeat-induced motions on the body surface by using a logarithmic transformation. However, the performance of the FFT-based method is deteriorated by both spectral variance and the natural variations in the pulse shape and IBI. To minimize spectral variance, we average over multiple contemporaneous range signals. To overcome the non-stationary nature of the heartbeat, we use a set of different FFT window lengths in parallel to compose a summary cepstrum. The proposed method was first developed for IBI extraction from multichannel BCG (covered by US patent 2010/0249628) [28]. In the current study, we adapt the method for the radar application.

**Figure 4:**
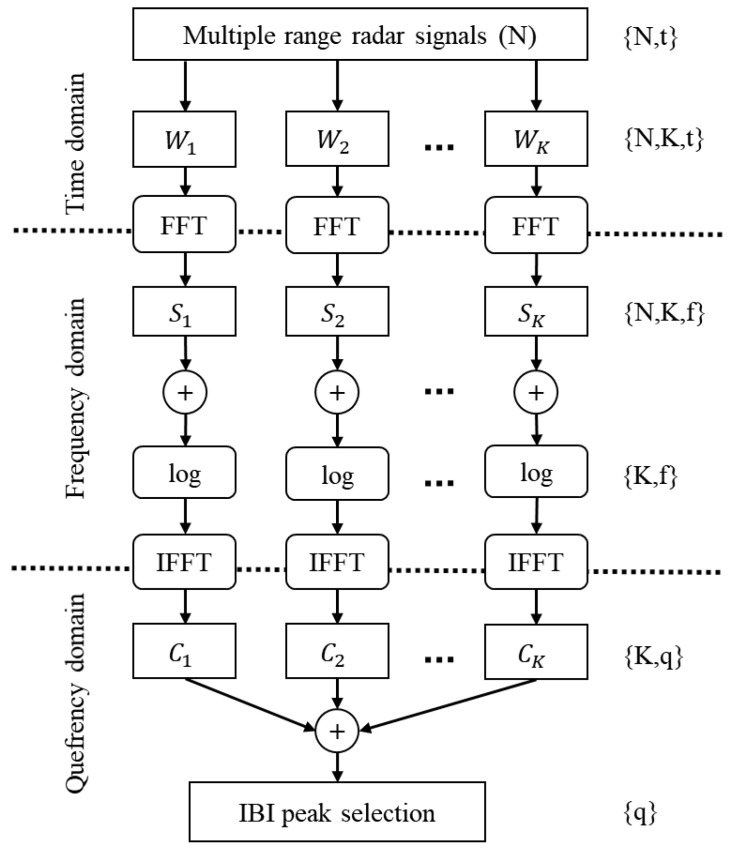
Interbeat interval extraction from a set of *N* range signals using *K* Fast Fourier Transform (FFT) windows. The process is repeated using overlapping FFT windows. The notations on the right represent the data dimensions at each phase; *t* denotes time, *f* frequency, and *q* the cepstrum lag time, or quefrency.

Figure 4 illustrates the radar IBI extraction process. We selected N=24 radar range bins to provide the input signals. Six different length Hamming windows $W_{i}$ (i=1,…,K, K=6) were used in parallel, each on every one of the *N* signals, to apply FFT and obtain the signal spectra. Each window was applied as a sliding window with strong overlap (0.1 s interval). The *K* window lengths ranged from 3.5 s to 20 s. The shortest windows were used to capture IBI approximately equal to half the window length, whereas the longest windows containing more than the optimal two heartbeats were used to detect IBI of rather constant pulse shape and interval. The FFT length was set to 40 s of samples for all windows and zero padding was used to improve resolution for the upcoming peak selection. To boost computational speed, the FFT length was rounded up to be divisible by sixteen.

Subsequently, the inverse FFT (IFFT) over the natural logarithm of the averaged spectra were computed to obtain the *K* cepstra. The cepstrum $C_{i}$ is defined as the real part of the inverse Fourier transform F−1{·} taken over the natural logarithm of an amplitude spectrum $|S_{i}|$

(1) $$C_{x}=real(F^{-1}\left\{log(\right|S_{x}\left|)\}\right)$$

as described in [27]. Whereas the spectrum $S_{i}$ contains peaks at the harmonic frequencies of the fundamental heartbeat frequency, in the cepstrum the harmonic spectral peaks appear as a single peak at the corresponding lag time, or quefrency [27].

The *K* cepstra are averaged to form the summary cepstrum. The overlaps between neighbouring cepstra were taken into consideration by applying a weighting window designed to produce equal sensitivity on each quefrency upon averaging.

Finally, the peaks in the summary cepstrum were taken as the IBI estimates. The quefrency resolution of the summary cepstrum and thus the IBI is directly the inverse of sampling frequency, and the slow-time resolution equals the sliding window interval. The peak selection was performed over the quefrency range from 0.5 s to 1.5 s in the cepstogram. It was initialized by taking the peaks that were strong with respect to both quefrency and time. Next, while weighting each initial IBI estimate by the corresponding peak height, a time averaged IBI $S_{IBI}$ was computed using a 60 s sliding Hamming window. Lastly, uncertain IBI were removed based on the cepstral peak height and distance to $S_{IBI}$; only the most prominent peaks were selected. The entire peak selection routine was repeated iteratively up to four times to allow $S_{IBI}$ to stabilize into the most prominent signal shape.

We note that our method can yield more than one estimates per actual interbeat interval. Thus, the average heart rate in the unit of beats per minute (bpm) was calculated by dividing 60 s by the average of the corresponding IBI estimates.

### 2.6. Heart Rate Variablity Analysis

HRV analysis employs a collection of features describing the beat-to-beat signal. The 13 time-domain features and 7 frequency domain features selected for this study are described in Table 2. In the context of frequency domain features, we chose to use the square root of power to rather present information scaled by the IBI signal amplitude than power itself. Welch’s method (30 s windows with trend removal, 75% window overlap) was used to estimate the power spectral density after resampling IBI to a constant 10 Hz sample frequency using cubic interpolation.

HRV features are commonly extracted from normal-to-normal peak intervals (NNI). Therefore, abnormal IBI were removed to obtain NNI estimates and replaced by linearly interpolated values [29]. A reference interval was considered abnormal if it changed more than 15% with respect to the previous one, whereas IBI from the radar were allowed a 20% change to account for the irregularity of the extracted IBI values.

### 2.7. Respiration Rate Extraction

As indicated in Figure 1, distinct methods were used to extract the respiratory vital sign from the reference devices and the radar. The reference respiration rate was derived from the reference signal through detrending and peak detection. The subtracted, smoothed trend was estimated using a 15 s Hann window, and the findpeaks function by MATLAB^®^ was used for peak detection (peak distances ranging from 1.4 s to 20 s were allowed). Local respiratory cycles were extracted from subsequent maxima and minima separately. Artefacts were identified based on the length and amplitude of the respiration cycle. Consecutive distorted cycles were combined when possible to better match the preceding and following five respiration cycles.

For the radar data, the respiratory motion was captured from the change of phase between consecutive chirps in the complex range profile, hereafter referred to as the phase signal. Specifically, the autocorrelation function (ACF) is applied on the phase signal of a selected range bin. The ACF has been previously proved to work in respiration monitoring on a single subject [15]. In this study, we aim for a robust implementation accurate for several subjects.

**Figure 5:**
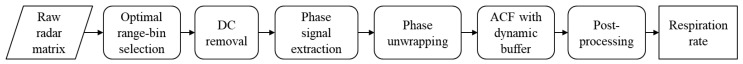
Respiration signal extraction.

The respiration rate extraction process is presented schematically in Figure 5. The optimal range bin corresponds to the distance where the primary target is located. The participants in this study were mostly stationary, which made it possible to select an optimal range-bin for each sub-measurement (row in Table 1). The range bin with the global maximum over the profiles in the range slow-time matrix was selected as the optimal range bin. Subsequently, the DC component was estimated globally from the full slow-time signal and removed. The slow-time profile in the optimal range bin was further used to extract an instantaneous phase signal, which was then unwrapped to remove ±2π phase jumps.

Because the phase signal closely follows periodic variation of the respiratory motion, we used the autocorrelation function to extract and quantify it. Unlike a periodogram, it can work with both long and short signals. The ACF at lag k<n can be written as

(2) $$R_{k}=\left[\sum_{i=1}^{n-k}\left(s_{i}-\mu \right)\left(s_{i+k}-\mu \right)\right]/\left[\sum_{i=1}^{n}{\left(s_{i}-\mu \right)}^{2}\right],$$

where the input sequences $\left[s_{1},s_{2},...,s_{n}\right]$ are generated by a sliding window function, and μ denotes their mean [30]. Given a suitable input sequence size, the lag of the maximum peak directly provides an estimate of the breathing interval. Thus, approximating one respiratory cycle as the average of 100 consecutive estimates, the window size was dynamically adjusted to contain 2.2 respiratory cycles.

Finally, post-processing focused on the removal of non-reliable estimates, such as outliers (over three standard deviations apart from the mean) and estimates with unstable phase due to other movements.

### 2.8. Performance Evaluation

Vital sign values (IBI or respiration interval) extracted from the radar data were each compared against the reference value closest in time. In all cases, a reference value resided within a maximum of 1.5 s temporal distance from the value.

Mean absolute error (MAE), root mean square error (RMSE), and Pearson correlation coefficient were selected for performance evaluation. While MAE is easy to interpret, RMSE emphasizes large errors, conveying information on where the most blatant errors occur. MAE and RMSE were computed individually for each participant and sub-measurement (row in Table 1). The resulting errors were weighted by the sub-measurement duration to aggregate representative error metrics for participants, activities, and lying positions. The aggregation methods are further described in Supplementary Material. For visual analysis, Bland-Altman plots were chosen to depict the agreement between the suggested methods and the reference. In contrast to correlation, the Bland-Altman plot describes both random and systematic error [31,32].

## 3. Results

The two sampling frequencies produced equally accurate results: the difference in RMSE was 0.001 s when measuring IBI and 0.169 1/min when measuring respiration rate. Thus, the measurements of either frequency are included in the remaining analysis.

The vital sign extraction results encompass ten participants. One of the 11 subjects (ID004) was excluded from the analysis due to unsuccessful data collection. Also, for three other participants (ID002, ID005, and ID010), the PSG respiration reference showed poor signal quality and was replaced with the secondary BCG-based reference. One participant (ID007) was excluded from the respiratory rate analysis due to poor quality reference in several sub-measurements.

### 3.1. Heart Rate

**Figure 6:**
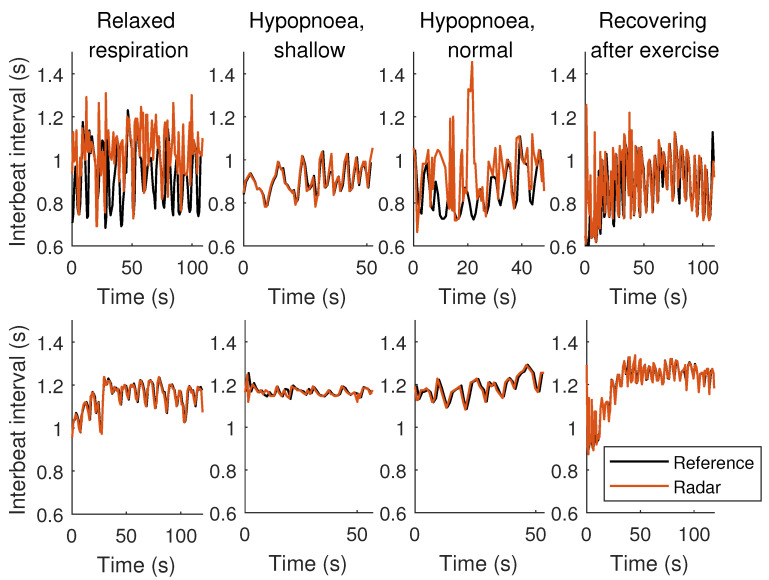
Interbeat interval samples of two participants (ID003 on the top, ID011 on the bottom) for each activity in the supine lying position.

The average measured interbeat interval over all measurements was 1.041 s (standard deviation SD 0.160 s, 5th percentile 0.820 s, 95th percentile 1.313 s), corresponding roughly 57.7 bpm, using the reference device. Respectively, the radar measured average was 1.053 s (SD 0.152 s, 5th percentile 0.832 s, 95th percentile 1.310 s), or roughly 57.0 bpm. Largest variations in the IBI were recorded when recovering from physical exercise; SD of 0.178 s was observed (0.164 s using the radar). Figure 6 illustrates samples of the extracted IBI signals with respect to the reference IBI for two participants. More samples are provided in the Supplementary Figure S1.

**Figure 7:**
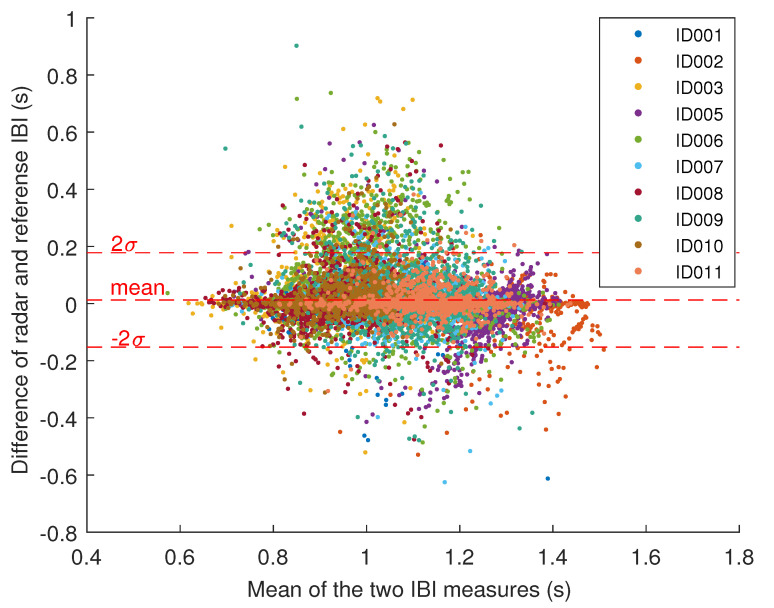
The Bland-Altman plot for interbeat interval. The dashed lines indicate the mean and the interval containing 95% of the samples.

Figure 7 illustrates the resulting differences between the radar and reference IBI in a Bland-Altman plot. The mean difference between the radar-derived and reference IBI is 0.013 s (SD 0.083 s), which roughly corresponds to a 0.71 bpm difference in the instantaneous heart rate.

Table 3 presents mean absolute error for each participant and activity. Differences between participants were below 0.07 s. To complement these results, we compensated for the varying number of heartbeat events per participant by taking an average not weighted by measurement duration. Also in this case, the participant MAE demonstrate differences under 0.07 s (and an overall average MAE of 0.038 s).

Considering MAE for each activity in Table 3, the error was at its largest when the participant was recovering after a short exercise session. Table 4 further describes the increase in MAE during the recovering activity as compared to the other activities measured in the same position. Additionally, Table 4 shows that differences between positions are in the order of milliseconds.

Considering all participants, activities, and positions, the IBI extracted from the radar exhibited an overall MAE of 0.038 s (SD 0.074 s, median absolute error 0.008 s) and RMSE of 0.084 s. The RMSE results presented in the Supplementary Tables S1 and S2 display similar trends as MAE. The Supplementary Figure S2 exemplifies the difference of the two metrics. Furthermore, the extracted IBI demonstrated a statistically significant Pearson correlation of 0.862 (*p*-value less than 0.01) as compared to the reference IBI. The IBI extracted from the radar are illustrated with respect to the reference values in the Supplementary Figure S4.

As for averaged heart rate in the units of beats per minute, MAE varied from 0.816 to 1.384 bpm for the different activities. These results are in line with the IBI results. Ranking the participant-wise errors, the order of some participants were reversed (e.g., ID003 and ID006), showing a small effect from the timestamp misalignment between the radar-derived and reference IBI. The mean absolute temporal distance between the two estimates was 0.24 s (SD 0.13 s), while maximum temporal misalignment was 0.74 s.

Overall, the heart rate analysis gave a MAE of 1.031 bpm, which corresponds to an average MAE of 0.016 s for the IBI. After removing ectopic beats, the results remained similar with an overall MAE of 1.079 bpm.

### 3.2. Heart Rate Variability

The HRV features were computed and evaluated for each sub-measurement individually. The results are summarized over all measurements in Table 5.

Most of the time-domain features exhibited notable correlation between the radar-derived and reference features. However, the MAE indicated notable 9–11% mean absolute errors in the pNNI20 and pNNI50. The remaining features agreed well with the reference features, exhibiting high correlation and small errors. For deviation-based features, MAE were lower than the standard deviation of the mean reference and for other features MAE was at most 5.5% (max HR) of the mean reference value.

Mean NNI, median NNI, and mean heart rate exhibited similar trends in terms of MAE between different participants as already observed in Table 3. Additionally, no large differences between neither the lying positions nor the activities were observed, although the recovering activity showed the largest error consistently.

The frequency-domain features showed mostly high correlations and low errors as well. The ⎷LF/HF ratio, LFnu, and HFnu features exhibited the most moderate correlations and the largest errors among the selected features. Yet, a 5% error in the normalized low or high frequency power may be considered acceptable. The errors for each activity, participant, or position did not seem to differ much for any of the frequency-domain features.

### 3.3. Respiration Rate

**Figure 8:**
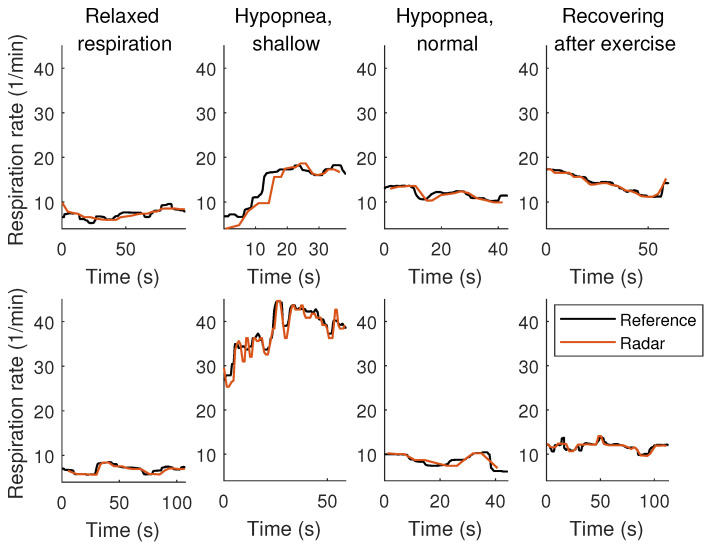
Respiration signal samples of two participants (ID003 on the top, ID011 on the bottom) for each activity in the supine lying position.

The measured respiration rates were on average 15.634 1/min (SD 7.492 1/min, 5th percentile 6.548 1/min, 95th percentile 32.535 1/min) for the reference devices. Correspondingly, the radar measured average was 15.855 1/min (SD 7.223 1/min, 5th percentile 7.138 1/min, 95th percentile 32.000 1/min). Most variation was recorded during the shallow respiration part of the hypopnoea simulation, showing SD of 10.010 1/min (9.097 1/min for the radar measured values). Samples of the extracted respiration rates are illustrated in Figure 8 with respect to the reference signals. More samples are depicted in the Supplementary Figure S3.

**Figure 9:**
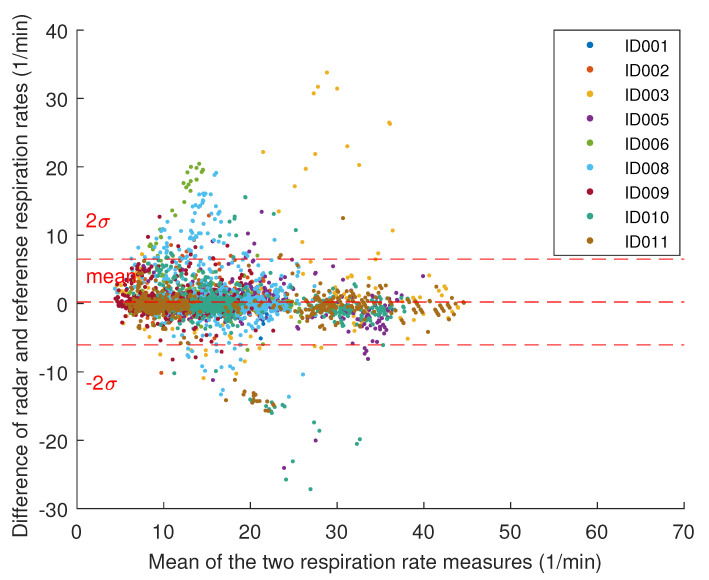
The Bland-Altman plot for respiration rate. The dashed lines indicate the mean and the interval containing 95% of the samples.

Figure 9 presents the Bland-Altman plot comparing respiration rates from the radar to those given by the reference devices. It exhibits a mean error of 0.221 breaths per minute (SD 3.137 1/min).

As presented in Table 6, the largest observed difference between participants in terms of MAE was 1.820 1/min. Additionally, when compensating for the different number of respiratory events per participant by averaging without weighting by measurement duration, the largest difference in participant MAE reduced to 1.487 1/min (with an overall average MAE of 1.354 1/min). For different activities, the smallest respiratory motions (during hypopnoea, shallow) exhibited the largest errors (especially ID003 and ID006).

In different positions, the differences in MAE are below 1.000 1/min, as presented in Table 7. However, the lateral measurement positions, especially the left lateral position, exhibited higher MAE as compared to the other two positions.

We obtained an overall MAE of 1.414 1/min (SD 2.810 1/min, median absolute error 0.515 1/min) and RMSE of 3.145 1/min for respiration rate. Detailed RMSE results are presented in the Supplementary Tables S3 and S4. Furthermore, the measurements exhibited a significantly high Pearson correlation of 0.910 (*p*-value less than 0.01), as visualized in the Supplementary Figure S5.

## 4. Discussion

The radar derived IBI tend to be slightly larger than the reference, although this systematic error varies considerably between participants (see Figure 7). Differences between participants in the normal ECG waveform, heart rate, and heart rate variability are all expected due to varying physiological factors. Thus, it comes as no surprise that the IBI of some participants may be more difficult to extract than that of others. The error in IBI for each participant is reasonable, in the order of tens of milliseconds.

As for different activities, the error in the extracted IBI was at its largest when a participant was recovering after an exercise session. This is when the largest respiratory motions are expected. Consistently, during the hypopnoea-mimicking shallow respiration, with close to no respiratory movement, the error was at its smallest. After the physical exercise, the participants were somewhat out of breath and it came naturally to take quick, deep breaths. Thus, the respiratory rate came closer to the expected range of the heart rate. As both motions are periodic, it became more difficult to distinguish the two. However, MAE for the recovering activity is only 0.052 s, thus showing good performance despite the challenging circumstances.

The overall error of 0.038 s indicates strong performance for the presented IBI extraction method in various scenarios. The different lying position did not affect the accuracy of the extracted IBI (see Table 4).

The presented results exceed previous achievements in heart rate monitoring obtained for lying positions. Anitori et al. presented an FFT method achieving a 10% error for heart rate, whereas we obtained 3.6% error for instantaneous heart rate [13]. Alizadeh et al. obtained a correlation of 80% for a single person, whereas we demonstrate an 86% correlation for ten participants [15]. Our results also compete with the results by Adib et al. who achieved a 99% median accuracy over a variety of measurement distances for participants in sitting positions [4]. At a similar distance of 2 m, they obtained a median accuracy of 98.7%, whereas our overall median absolute error of 0.008 s corresponds to a 99.2% accuracy and the mean absolute error of 0.038 s corresponds to 96.3% accuracy.

In HRV analysis, most time and frequency-domain features obtained from the IBI estimates demonstrated high agreement with the reference values. The pNNI20 and pNNI50 time-domain features were the notable exceptions. The error for these parameters presumably followed from the numerous estimates for each actual heartbeat event given by our IBI extraction method, which shifts the number of intervals exceeding 20 or 50 ms as compared to the total number of intervals. The extra estimates might have also affected the error in minimum and maximum heart rates. Yet, many HRV parameters remain useful when extracted from the radar.

As for respiratory rate, smallest respiratory motions were the most difficult to detect using the radar (see Table 6). Yet, the average MAE remained comparable to that of normal respiration (1.222 1/min), supporting that the method is reliable in various real-life scenarios.

Respiration rate extraction was found to be more complicated in lateral positions; the highest error was measured in one of the two positions for all expect one participant. Notably, two participants (ID003 and ID006) exhibited exceptionally high error (especially RMSE) in one of the lateral positions during the shallow respiration period of the hypopnoea simulation, contributing notably to the overall error.

The observed differences in the error of the two lateral positions may be tracked back to the measurement setting. The RIP belt used to measure the primary respiration reference is an elastic band around the participants thorax; the change of posture could loosen the RIP belt by sliding it from its original location. As described in the protocol (Table 1), the participants always visited the right lateral position before the left, which may have resulted in larger error in the left lateral position. Additionally, the error may be higher in lateral positions as compared to the other two because of the smaller prevalence of the respiratory motion in the observed area.

Altogether, our respiration extraction method is comparable to the state-of-the-art methods. As compared to Alizadeh et al. who used a similar autocorrelation approach to extract respiration with 94% correlation with the reference, we expand the method for several participants and achieve a 91% correlation. Adib et al. on the other hand achieved a 99.4% median accuracy in seated positions, while we show an overall 96.5% median absolute accuracy and a 91.0% mean absolute accuracy in lying positions [4]. However, in contrast to previous studies, our study considered a wide range of respiration rates including breathing with minimal motion [4,13,14,15]. Despite the challenging setup, our method performed robustly in the different scenarios. The high correlation between the radar-extracted and reference estimates is a remarkable result given the wide range of recorded respiratory motions.

The chosen methods of cepstrum for IBI extraction and autocorrelation for respiration rate extraction are closely related, as both can be formulated as an inverse Fourier transform from the power spectra [33]. Cepstral analysis emphasizes the harmonic frequencies of a spectrum. The signal power of rapid bursts, such as heartbeats, is mainly carried by the harmonic spectral peaks, which are further emphasized in the logarithm of the spectrum. In contrast to heartbeats, the signal power of respiratory motion is concentrated on the base frequency, making autocorrelation a suitable approach to extract respiration [33].

The presented vital sign extraction methods are limited with respect to real time applications. The IBI extraction requires a delay equal to the longest FFT window (20 s) in addition to the delay due to the iterative smoothing to remove uncertain estimates (upto 4 min). The IBI extraction performance could however be improved if it was implemented in parallel with another method, such as the data fusion method described in [34]. The respiration rate extraction is restricted by the maximum delay equal to the maximum ACF peak extraction buffer (default of 15 s). Furthermore, the optimal range bin selection and DC removal were computed globally for each sub-measurement, and would need to be performed adaptively to account for changes of position during sleep.

The presented results are limited by the restricted set of participants and thus the methods may not generalize as well for broader groups. Although data collection in a controlled environment allowed us to capture a wide range of vital signs, the natural next step would be to test the presented methods on a large study group in over-night measurements. The suggested techniques could also be optimized for personal vital sign patterns to improve performance for individuals. For future work, we note that while here the clean reference ECG enabled the use of the standard MATLAB^®^ tool findpeaks, the Pan-Tompkins algorithm is suggested for reference R peak extraction. In addition, the results were obtained on subjects lying still and therefore do not directly apply to moving subjects. However, applying noise removal prior to the vital sign extraction methods could increase performance for moving subjects.

## 5. Conclusions

Our results suggest that the cost-effective 24 GHz FMCW radar together with the proposed vital sign extraction methods represent a solution that can deliver accurate results for nocturnal vital sign monitoring even during various conditions, such as sleep apnoea. We obtained state-of-the-art level accuracies for heart rate monitoring while, to the best of our knowledge, being the first to report as low errors in recording instantaneous interbeat intervals using a similar device [4]. Moreover, we demonstrated the radar’s feasibility in heart rate variability analysis. Finally, we presented remarkably accurate results in respiration monitoring, maintaining a reasonable error level from abnormally shallow respiration to high-volume gasping. As far as we know, this is the first study to include uncommonly small respiratory motions in the studied respiratory range and evaluate the FMCW radar technology for apnoea indication.

While our study focused on nocturnal vital sign monitoring applications, the technology can be applicable to various other purposes where the subjects remain mainly still, such as monitoring bedridden patients or the elderly, or finding victims trapped under constructions at disaster scenes. In the future, the methods can be tested on authentic nocturnal measurements and adjusted for more advanced 60 GHz radars, enabling the measurement of multiple subjects simultaneously, despite close proximity [35]. Other remaining challenges include, e.g., decreasing the effect of motion artefacts and reducing the time delay during signal extraction to enable real-time applications.

## Figures and Tables

**Table 1:** Vital sign measurement protocol.

Activity	Position	Duration (min)	
		110 Hz	154 Hz
Relaxed respiration	Supine	2	2
Relaxed respiration	Right lateral	2	2
Relaxed respiration	Prone	2	2
Relaxed respiration	Left lateral	2	2
Relaxed respiration	Supine	2	2
Hypopnoea simulation, shallow respiration	Supine	1	-
Hypopnoea simulation, normal respiration	Supine	1	-
Hypopnoea simulation, shallow respiration	Right lateral	1	-
Hypopnoea simulation, normal respiration	Right lateral	1	-
Hypopnoea simulation, shallow respiration	Prone	1	-
Hypopnoea simulation, normal respiration	Prone	1	-
Hypopnoea simulation, shallow respiration	Left lateral	1	-
Hypopnoea simulation, normal respiration	Left lateral	1	-
Recovering after exercise ${}^{a}$	Supine	2	2
Total measurement time (min)		20	12

${}^{a}$ Preceded by a two-minute exercise (not measured).

**Table 2:** Heart rate variability features explained.

Domain	Feature	Description
	Mean NNI	Average over all normal-to-normal peak intervals (NNI)
	Median NNI	Median over all NNI
	RMSSD	Root mean square of consecutive differences of adjacent NNI
	SDSD	Standard deviation of consecutive differences of adjacent NNI
	SDNN	Standard deviation of NNI
	CVNNI	Coefficient of variation
Time	CVSD	Coefficient of variation for successive differences
	pNN20	Percentage of interval differences exceeding 20 ms
	pNN50	Percentage of interval differences exceeding 50 ms
	HR	Heart rate
	STD${}_{HR}$	Standard deviation of heart rate
	Min HR	Minimum heart rate
	Max HR	Maximum heart rate
	⎷Total power	Square root of total power
	⎷VLF	Square root of very low frequency power (0.0033–0.04 Hz)
	⎷LF	Square root of low frequency power (0.4–0.15 Hz)
Frequency	⎷HF	Square root of high frequency power (0.15–0.4 Hz)
	⎷LF/HF ratio	Square root of the ratio of low and high frequency power
	LFnu	Normalized low frequency power
	HFnu	Normalized high frequency power

**Table 3:** Mean absolute error (MAE, s) for interbeat intervals with respect to activity and participant.

Participant ID	Relaxed	Hypopnoea, Shallow	Hypopnoea, Normal	Recovering	Participant MAE
ID001	0.016	0.013	0.011	0.018	0.015
ID002	0.040	0.015	0.028	0.050	0.034
ID003	0.068	0.032	0.067	0.058	0.061
ID005	0.029	0.032	0.023	0.095	0.038
ID006	0.073	0.074	0.061	0.116	**0.077**
ID007	0.023	0.010	0.057	0.027	0.027
ID008	0.041	0.019	0.029	0.032	0.036
ID009	0.046	0.009	0.090	0.061	0.051
ID010	0.023	0.032	0.020	0.019	0.023
ID011	0.018	0.014	0.017	0.020	0.018
Activity MAE	0.037	0.026	0.042	**0.052**	0.038 ${}^{a}$

The largest activity and participant MAEs are bolded. ${}^{a}$ The total MAE over all activities and participants

**Table 4:** Mean absolute error (s) for interbeat intervals with respect to activity and lying position.

Position	Relaxed	Hypopnoea, Shallow	Hypopnoea, Normal	Recovering ${}^{a}$	Position MAE
Supine	0.034	0.014	0.042	0.052	0.038 ${}^{b}$
Right lateral	0.040	0.036	0.041	-	0.040
Prone	0.039	0.028	0.054	-	**0.040**
Left lateral	0.038	0.028	0.032	-	0.035
Activity MAE	0.037	0.026	0.042	**0.052**	0.038 ${}^{c}$

The largest mean MAEs are bolded. ${}^{a}$ Recovering was only measured in the supine position. ${}^{b}$0.033 s if the recovering activity is not considered. ${}^{c}$ The total MAE over all activities and positions.

**Table 5:** Comparison of the heart rate variability features.

Feature	Mean ± Standard Deviation		MAE	Correlation
	Radar	Reference		
Time-domain features				
Mean NNI	1.06 ± 0.13	1.05 ± 0.14	0.02	0.98
Median NNI	1.06 ± 0.14	1.05 ± 0.15	0.02	0.98
RMSSD	0.05 ± 0.02	0.04 ± 0.02	0.01	0.81
SDNN	0.07 ± 0.03	0.07 ± 0.04	0.01	0.88
SDSD	0.05 ± 0.02	0.05 ± 0.02	0.01	0.81
CVNNI	0.07 ± 0.03	0.07 ± 0.04	0.01	0.89
CVSD	0.05 ± 0.02	0.04 ± 0.02	0.01	0.84
pNNI20	50.59 ± 17.75	50.92 ± 25.88	11.12	0.81
pNNI50	28.10 ± 15.03	25.31 ± 21.08	9.19	0.84
Mean HR	57.68 ± 7.06	58.52 ± 7.66	1.15	0.97
STD${}_{HR}$	4.03 ± 1.93	4.09 ± 2.44	0.77	0.86
Min HR	48.87 ± 5.95	51.03 ± 6.67	2.61	0.87
Max HR	70.24 ± 9.79	70.87 ± 12.02	3.93	0.79
Frequency-domain features				
⎷Total power	0.09 ± 0.04	0.09 ± 0.04	0.01	0.95
⎷VLF	0.04 ± 0.02	0.05 ± 0.02	4.9 $\times 10^{-3}$	0.94
⎷LF	0.07 ± 0.03	0.07 ± 0.03	0.01	0.93
⎷HF	0.04 ± 0.01	0.04 ± 0.02	0.01	0.86
⎷LF/HF ratio	1.58 ± 0.37	1.66 ± 0.41	0.20	0.72
LFnu	68.94 ± 13.27	70.70 ± 13.28	5.44	0.78
HFnu	31.06 ± 13.27	29.30 ± 13.28	5.44	0.78

**Table 6:** Mean absolute error for respiration rates with respect to activity and participant.

Participant ID	Relaxed	Hypopnoea, Shallow	Hypopnoea, Normal	Recovering	Participant MAE
ID001	0.367	0.725	0.418	0.251	0.487
ID002	0.940	1.694	1.124	0.611	1.088
ID003	1.305	5.118	0.505	0.339	2.272
ID005	0.751	2.419	0.934	0.617	1.075
ID006	0.856	3.852	0.369	0.536	1.331
ID008	2.626	2.310	1.601	1.170	**2.308**
ID009	1.049	2.610	0.869	1.833	1.336
ID010	0.737	2.516	0.752	3.094	1.392
ID011	1.311	1.155	0.981	0.388	1.114
Activity MAE	1.222	**2.408**	0.887	1.149	1.414 ${}^{a}$

The largest activity and participant MAEs are bolded. ${}^{a}$ The total MAE over all activities and participants.

**Table 7:** Mean absolute error for respiration rates with respect to activity and lying position.

Position	Relaxed	Hypopnoea, Shallow	Hypopnoea, Normal	Recovering ${}^{a}$	Position MAE
Supine	1.002	1.270	1.149	1.149	1.086 ${}^{b}$
Right lateral	1.096	3.588	0.682	-	1.656
Prone	1.256	1.458	0.707	-	1.225
Left lateral	1.732	3.454	1.001	-	**2.064**
Activity MAE	1.222	**2.408**	0.887	1.149	1.414 ${}^{c}$

The largest mean MAEs are bolded. ${}^{a}$ Recovering was only measured in the supine position. ${}^{b}$1.061 1/min if the recovering activity is not considered. ${}^{c}$ The total MAE over all activities and positions.

## Supplementary Materials

The following are available online at https://www.mdpi.com/1424-8220/20/22/6505/s1; Aggregated error metrics, Figure S1: Examples of interbeat intervals extracted for each subject during relaxed respiration in the supine lying position, Table S1: Root mean square error for interbeat intervals with respect to activity and participant, Table S2: Root mean square error for interbeat intervals with respect to activity and lying position, Figure S2: Example of interbeat intervals extracted from an arrhythmic sequence, Figure S3: Examples of respiration signals for each subject during relaxed respiration in the supine lying position, Table S3: Root mean square for respiration rates with respect to activity and participant, Table S4: Root mean square error for respiration rates with respect to activity and lying position, Figure S4: Correlation of the interbeat interval derived from the radar signal and the reference IBI, Figure S5: Correlation of the respiration rate derived from the radar signal and the reference respiration rates.
