# Supplementary material for: Vital Sign Monitoring Using FMCW Radar in Various Sleeping Scenarios
Source: Sensors (Basel). 2020 Nov 14;20(22):6505. doi: 10.3390/s20226505 (PMC7696080; doi:10.3390/s20226505)
Supplement: Supplementary file 1 [file sensors-20-06505-s001.pdf]

Supplementary Materials: Vital sign monitoring using FMCW radar in various sleeping scenarios

Emmi Turppa [1,*], Juha M. Kortelainen [1], Oleg Antropov [1] and Tero Kiuru [1]

1. Aggregated error metrics

The performance metrics that measure error were computed for each 2-minute measurement independently. When the error measures were combined to compare the total errors between entities, such as participants or different activities, each measured instance was considered equal to others. Thus, the total mean absolute error (MAE) in a set of measurements was calculated as an average MAE weighted with the measurement lengths. In contrast, to couple the MAE with a valid total standard deviation of error s over N measurements, the problem was formulated as

$$s_{total} = \sqrt{\frac{\sum_{n=1}^{N}((w_n - 1) * s_n^2 + w_n * (ME_n - \mu^2))}{\sum_{n=1}^{N}(w_n - 1)}}, \quad (1)$$

where w_n is the length of the nth measurement, s_n its standard deviation, ME its mean error, and μ is the total mean error of the N measurements.

Additionally, the total root mean squared error (RMSE) over N measurements was computed with

$$RMSE_{total} = \sqrt{\frac{\sum_{n=1}^{N} w_n * RMSE_n^2}{\sum_{n=1}^{N} w_n}}, \quad (2)$$

where $RMSE_n^2$ is the mean squared error of the nth measurement.

2. Examples of interbeat intervals for each subject

Figure S1 presents interbeat interval samples for each subject during relaxed respiration in the supine lying position.

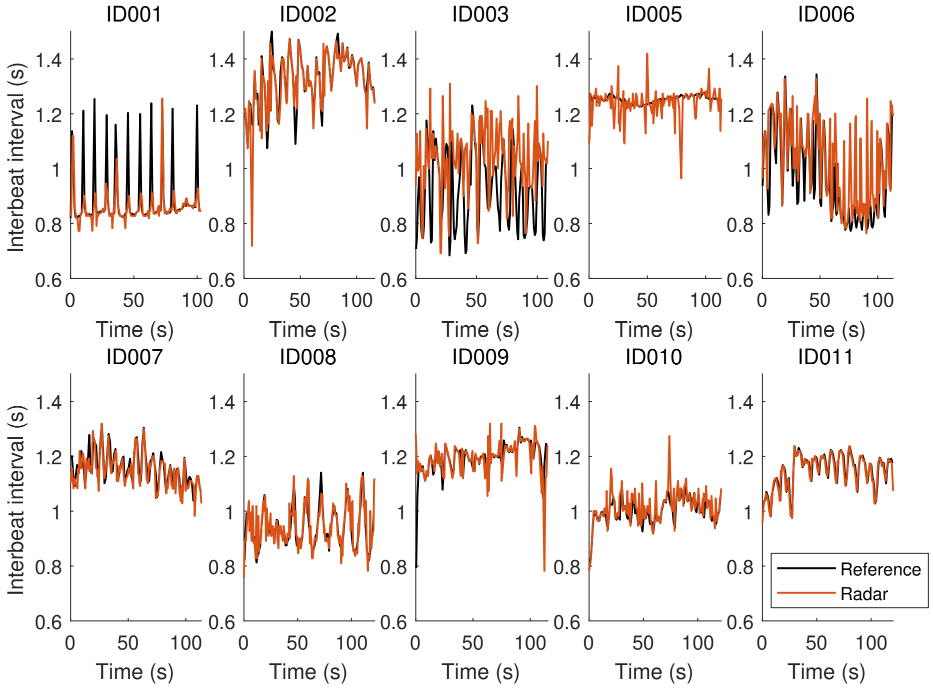

Figure S1. Examples of interbeat intervals extracted for each subject during relaxed respiration in the supine lying position.

3. RMSE of the interbeat interval estimates

The root mean square errors for each participant and for each lying position with respect to each activity are presented in Tables S1 and S2, respectively. The RMSE results agree well with the corresponding results using MAE, exhibiting similar patterns with respect to participants and activities, while the differences between positions remain trivial.

Table S1. Root mean square error for interbeat intervals with respect to activity and participant

Participant ID	Relaxed	Hypopnoea, shallow	Hypopnoea, normal	Recovering	Participant RMSE
ID001	0.049	0.042	0.034	0.028	0.043
ID002	0.085	0.051	0.055	0.095	0.076
ID003	0.135	0.069	0.125	0.121	0.123
ID005	0.057	0.072	0.047	0.160	0.081
ID006	0.128	0.119	0.104	0.181	**0.132**
ID007	0.043	0.034	0.107	0.041	0.055
ID008	0.082	0.046	0.072	0.061	0.076
ID009	0.090	0.030	0.141	0.107	0.098
ID010	0.053	0.075	0.044	0.038	0.054
ID011	0.037	0.034	0.036	0.046	0.038
Activity RMSE	0.081	0.065	0.088	**0.105**	0.084[a]

The largest activity and participant RMSEs are bolded.
[a]The total RMSE over all activities and participants.

Table S2. Root mean square error for interbeat intervals with respect to activity and lying position

Position	Relaxed	Hypopnoea, shallow	Hypopnoea, normal	Recovering[a]	Position RMSE
Supine	0.077	0.037	0.097	0.105	**0.086**[b]
Right lateral	0.089	0.079	0.079	-	0.085
Prone	0.082	0.064	0.101	-	0.083
Left lateral	0.080	0.071	0.072	-	0.077
Activity RMSE	0.081	0.065	0.088	**0.105**	0.084[c]

The largest mean RMSEs are bolded.
[a]Recovering was only measured in the supine position.
[b] 0.077 seconds if the recovering activity is not considered.
[c]The total RMSE over all activities and positions.

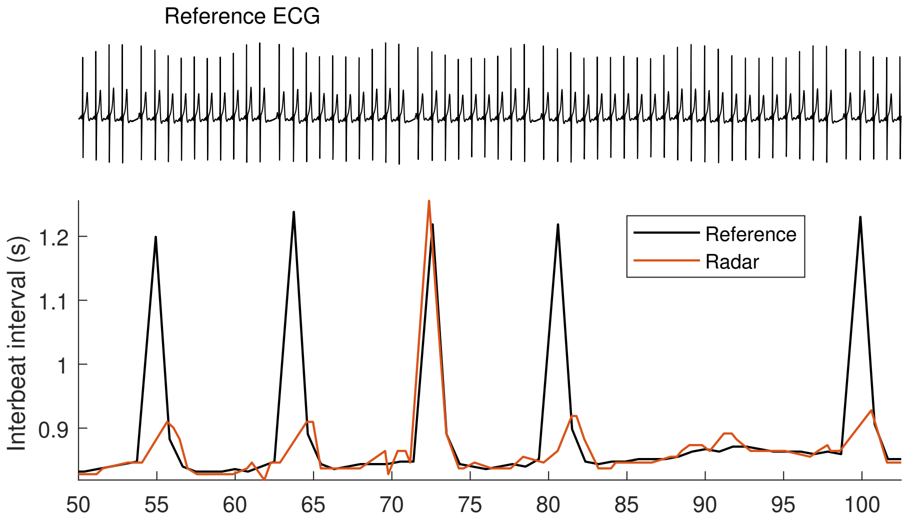

Figure S2. Example of interbeat intervals extracted from an arrhythmic sequence, extracted from both the reference ECG (visualized at the top) and the radar.

As opposed to MAE, the RMSE emphasizes large errors, indicating where the largest errors occur. In this case, most of the largest errors took place in the measurements that also exhibited the largest MAE values. This implies that the radar IBI extraction performs in a stable manner in the various scenarios.

It is additionally noted that MAE is more robust to possible arrhythmia episodes, during which there are no beats for the radar to detect. Figure S2 illustrates an arrhythmic ECG sequence together with the interbeat intervals extracted from both the reference ECG and the radar. In the ECG, the arrhythmia is visible as elongated gaps between the otherwise regularly periodic R peaks, whereas in the IBI extracted from the reference, they are visible as peaks. The significant yet relatively rare points of high error between the radar and reference IBI emphasize the root mean square error, while the mean absolute error is not affected as much. The mean absolute error in the illustrated sequence was 0.013 s and the root mean square error 0.042 s.

4. Examples of respiration rates for each subject

Figure S3 presents respiration signal samples for each subject during relaxed respiration in the supine lying position.

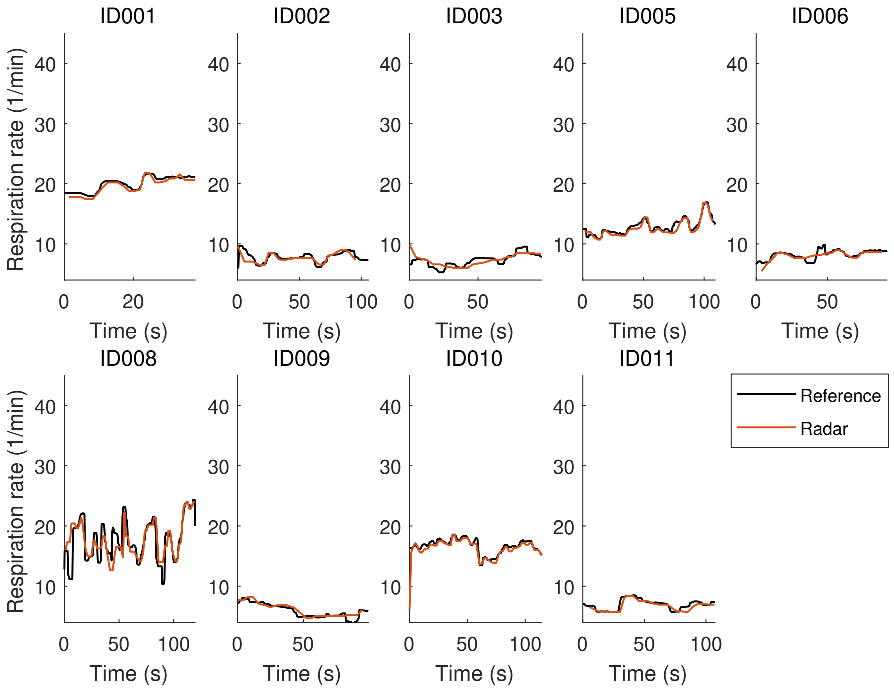

Figure S3. Examples of respiration signals for each subject during relaxed respiration in the supine lying position.

5. RMSE of the respiration rate estimates

Tables S3 and S4 present the RMSE when comparing the radar respiration rates to those given by the reference devices. Similarly as with MAE, the hypopnoea simulation (shallow respiration) with small respiratory motion was more difficult to detect accurately than the larger motions. As for the participants, ID003 had the largest MAE. Comparing with the MAE results, this implies that ID003 had larger error in individual samples, or that there are more outlier-like values. In addition, the same trend of increased error in lateral positions could again be observed.

Further analysis revealed that there were two measurements with exceptionally high RMSE. Both occurred during the shallow respiration period of the hypopnoea simulation; ID003 showed an RMSE

Table S3. Root mean square error for respiration rates with respect to activity and participant

Participant ID	Relaxed	Hypopnoea, shallow	Hypopnoea, normal	Recovering	Participant RMSE
ID001	0.582	1.001	0.532	0.322	0.728
ID002	1.638	2.767	2.065	0.855	1.933
ID003	2.112	9.145	0.742	0.459	**5.371**
ID005	1.401	3.714	1.669	0.890	2.048
ID006	1.852	7.531	0.480	1.058	3.587
ID008	4.559	3.359	2.687	1.794	4.061
ID009	1.794	3.699	1.568	2.853	2.264
ID010	1.365	5.298	1.274	5.505	3.283
ID011	3.322	1.779	1.878	0.545	2.548
Activity RMSE	2.609	**4.954**	1.683	2.547	3.145[a]

The largest activity and participant RMSEs are bolded.
[a]The total RMSE over all activities and participants.

Table S4. Root mean square error for respiration rates with respect to activity and lying position

Position	Relaxed	Hypopnoea, shallow	Hypopnoea, normal	Recovering[a]	Position RMSE
Supine	1.924	2.144	2.085	2.547	2.154[b]
Right lateral	2.244	6.767	1.269	-	3.836
Prone	2.799	2.322	1.190	-	2.486
Left lateral	3.670	6.694	1.957	-	**4.489**
Activity RMSE	2.609	**4.954**	1.683	2.547	3.145[c]

The largest mean RMSEs are bolded.
[a]Recovering was only measured in the supine position.
[b] 1.978 1/min if the recovering activity is not considered.
[c]The total RMSE over all activities and positions.

of 14.728 1/min in the left lateral position, whereas ID006 demonstrated an RMSE of 17.726 1/min in the right lateral position. While these individual cases affected the aggregated RMSE values, the highest RMSE was measured in one of the lateral positions for all except one participant.

6. Estimate correlation

Figure S4 and S5 depict the relationship between individual radar-measured and reference values. Figure S4 illustrates the relation between interbeat interval values (IBI) and Figure S5 the relation between respiration rates.

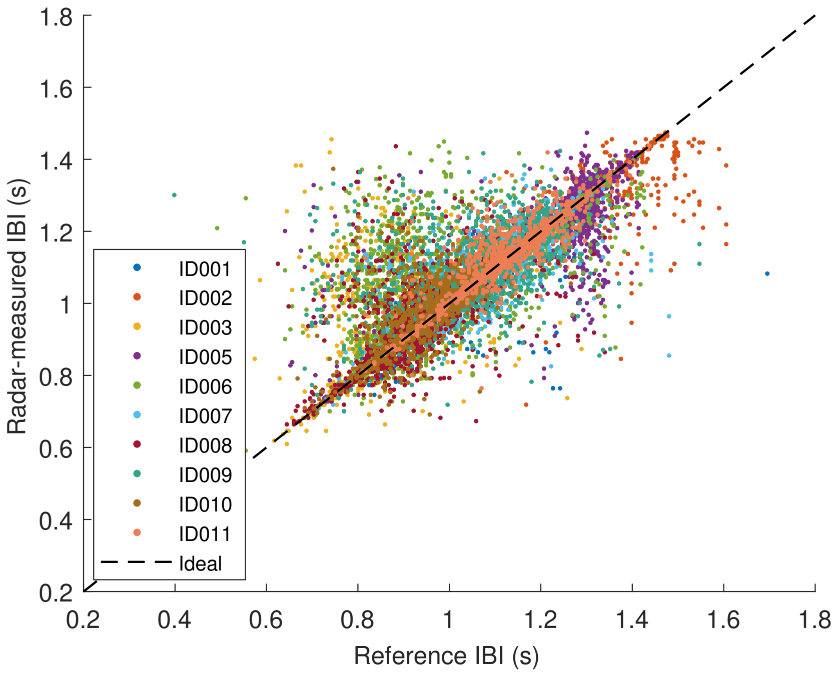

Figure S4. The interbeat interval derived from the radar signal presented against the corresponding reference IBI.

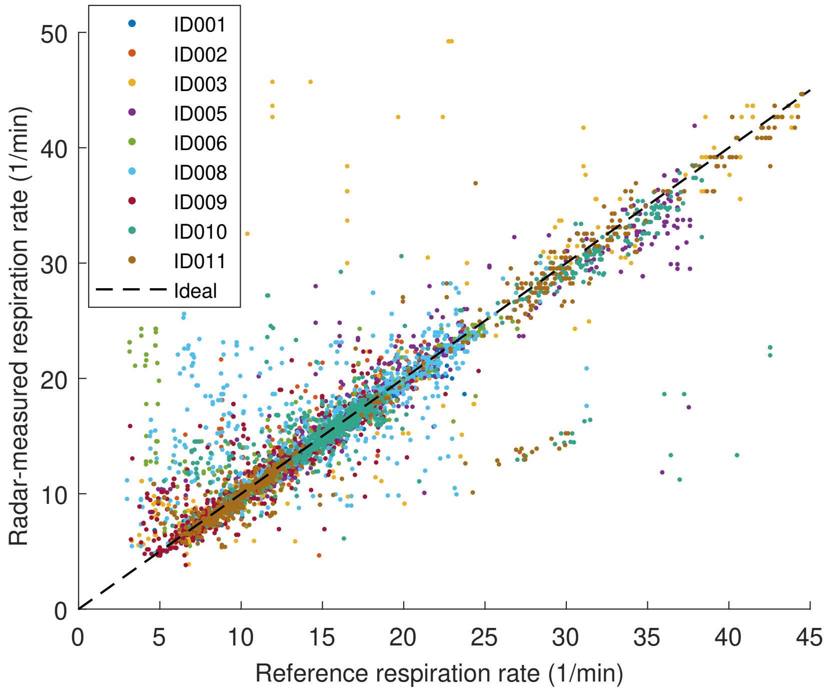

Figure S5. The respiration rate derived from the radar signal presented against the corresponding reference respiration rates.
